# Metabolic engineering *Saccharomyces cerevisiae* for de novo production of the sesquiterpenoid (+)-nootkatone

**DOI:** 10.1186/s12934-020-1295-6

**Published:** 2020-02-03

**Authors:** Xiangfeng Meng, Hui Liu, Wenqiang Xu, Weixin Zhang, Zheng Wang, Weifeng Liu

**Affiliations:** State Key Laboratory of Microbial Technology, Shandong University, No. 72 Binhai Road, Qingdao, 266237 People’s Republic of China

**Keywords:** (+)-Valencene, (+)-Nootkatone, *Saccharomyces cerevisiae*, Sesquiterpene, Dehydrogenases

## Abstract

**Background:**

(+)-Nootkatone is a highly valued sesquiterpenoid compound, exhibiting a typical grapefruit aroma and various desired biological activities for use as aromatics and pharmaceuticals. The high commercial demand of (+)-nootkatone is predominately met by chemical synthesis, which entails the use of environmentally harmful reagents. Efficient synthesis of (+)-nootkatone via biotechnological approaches is thus urgently needed to satisfy its industrial demand. However, there are only a limited number of studies that report the de novo synthesis of (+)-nootkatone from simple carbon sources in microbial cell factories, and with relatively low yield.

**Results:**

As the direct precursor of (+)-nootkatone biosynthesis, (+)-valencene was first produced in large quantities in *Saccharomyces cerevisiae* by overexpressing (+)-valencene synthase CnVS of *Callitropsis nootkatensis* in combination with various mevalonate pathway (MVA) engineering strategies, including the expression of CnVS and farnesyl diphosphate synthase (ERG20) as a fused protein, overexpression of a truncated form of the rate-limiting enzyme 3-hydroxy-3-methylglutaryl-CoA (HMG-CoA) reductase (tHMG1), and downregulating the squalene synthase enzyme (ERG9). These approaches altogether brought the production of (+)-valencene to 217.95 mg/L. Secondly, we addressed the (+)-valencene oxidation by overexpressing the *Hyoscyamus muticus* premnaspirodiene oxygenase (HPO) variant (V482I/A484I) and cytochrome P450 reductase (ATR1) from *Arabidopsis thaliana*. However, (+)-valencene was predominantly oxidized to β-nootkatol and only minor amounts of (+)-nootkatone (9.66 mg/L) were produced. We further tackled the oxidation of β-nootkatol to (+)-nootkatone by screening various dehydrogenases. Our results showed that the short-chain dehydrogenase/reductase (SDR) superfamily dehydrogenases ZSD1 of *Zingiber zerumbet* and ABA2 of *Citrus sinensis* were capable of effectively catalyzing β-nootkatol oxidation to (+)-nootkatone. The yield of (+)-nootkatone increased to 59.78 mg/L and 53.48 mg/L by additional overexpression of ZSD1 and ABA2, respectively.

**Conclusion:**

We successfully constructed the (+)-nootaktone biosynthesis pathway in *S. cerevisiae* by overexpressing the (+)-valencene synthase CnVS, cytochrome P450 monooxygenase HPO, and SDR family dehydrogenases combined with the MVA pathway engineering, providing a solid basis for the whole-cell production of (+)-nootkatone. The two effective SDR family dehydrogenases tested in this study will serve as valuable enzymatic tools in further optimizing (+)-nootkatone production.

## Background

Sesquiterpenes are a large group of terpenoids and generally found in the plant essential oils [1, 2]. Many of them show a unique odor with a very low perception threshold, which draws intense interest from the beverage, cosmetic, and medical industries [1, 2]. The oxidized sesquiterpene (+)-nootkatone exhibits a typical grapefruit aroma at a very low threshold of about 1 μg/L [3, 4]. Notably, (+)-nootkatone has also been reported to show interesting therapeutic potentials, such as anticancer, antiplatelet aggregation, antimicrobial, and anti-inflammation activities, and thus represents a promising drug precursor [3, 4]. (+)-Nootkatone was initially isolated from the heartwood of Alaska Yellow Cedar and was later also found to be constituent of essential oils from grapefruits and pummelo [4]. However, (+)-nootkatone is only present in trace amounts in these plants and natural extraction is proven to be very inefficient, limiting its commercial applications. Although chemical oxidation of (+)-valencene has been employed to produce (+)-nootkatone to satisfy the high industry demand, they involve the use of environment-unfriendly oxidizing reagents such as tert-butyl peracetate, tert-butyl hydroperoxide, or heavy metals [5, 6]. With the development of synthetic biology, constructing microbial cell factories represents a promising alternative for the production of (+)-nootkatone [1].

The common precursor for terpenoid synthesis is isopentenyl diphosphate (IPP), which is derived from the mevalonate pathway (MVA) or the methyl-d-erythritol phosphate (MEP) pathway [7–9]. Condensation of IPP and its isomers dimethylallyl pyrophosphate (DMAPP) results in geranyl pyrophosphate (GPP) and farnesyl diphosphate (FPP), which are the precursors for monoterpene and sesquiterpene synthesis, respectively [1, 8]. *Saccharomyces cerevisiae* naturally synthesizes FPP through the MVA pathway and has thus been used for enhanced production of industrially relevant terpenoids by introducing the corresponding heterologous terpene synthase in combination with several metabolic engineering approaches [1]. The synthesized terpene can be further modified by regio- and stereo-specific oxidation, reduction, and acetylation et al., generating structurally diverse terpenoid compounds [1, 10].

As the direct precursor of (+)-nootkatone, ample supply of (+)-valencene is a prerequisite for efficient (+)-nootkatone synthesis [4, 10]. Up to now, only several (+)-valencene synthases have been identified and tested for (+)-valencene biosynthesis, including VvVal of *Vitis vinifera* [11], Cstps1 of *Citrus sinensis* [12], GFTpsD of *Citrus* × *paradis* [13], and CnVS of *C. nootkatensis* [13]. Among others, CnVS is proven to be the most robust one regarding catalytic pH and temperature, which is a desired property for the application in different hosts or under various physiological conditions [13]. Overexpression of CnVS in yeast strain WAT11, however, gave rise to only 1.36 mg/L of (+)-valencene [13]. (+)-Valencene production up to 3 mg/L has been achieved by expressing the (+)-valencene synthase GFTpsD and simultaneously downregulating the squalene synthase in *S. cerevisiae* [14]. Apart from yeast, (+)-valencene production has also been attempted in *Corynebacterium glutamicum* [15] and *Schizophyllum commune* [16], yielding 2.41 mg/L and 16 mg/L valencene, respectively. Thus, the yield of (+)-valencene in different hosts is still too low to reach the industrial demand, hindering its further oxidation for (+)-nootkatone biosynthesis.

The biosynthesis of (+)-nootkatone has been described by either oxidizing the exogenously added (+)-valencene by whole-cell catalysts or simultaneous expression of the (+)-valencene oxidases in (+)-valencene production strains [4]. Several cytochrome P450 enzymes, including CYP109B1 of *Bacillus subtilis* [17], CYP71D51v2 of tobacco [10], CYP71D4 of *Solanum tuberosum* [10], CYP71AV8 of *Cichorium intybus* [18], and the premnaspirodiene oxygenase of *Hyoscyamus muticus* (CYP71D55, HPO) [19] have been overexpressed in yeast and employed as whole-cell catalysts to catalyze the oxidation of (+)-valencene. Further enzyme engineering of HPO targeting its substrate recognition site has identified an HPO variant (HPO V482I/A484I) with a fivefold improved catalytic efficiency in the oxidation of (+)-valencene. However, these P450 enzymes mostly generate β-nootkatol as the predominant product and produce only minor amounts of (+)-nootkatone [18, 20]. On the other hand, a lipoxygenase ValOx from *Pleurotus sapidus* was found to catalyze the oxidation of (+)-valencene primarily to (+)-nootkatone [21, 22], although it has not been explored further for its applicability in (+)-nootkatone biosynthesis in a microbial cell factory. More recently, an alcohol dehydrogenase from *Pichia pastoris* has been found to be capable of converting β-nootkatol to (+)-nootkatone [23]. Nevertheless, efficient and selective oxidation of (+)-valencene to nootakatone is still challenging, which represents another bottleneck in (+)-nootkatone biosynthesis to meet the high industrial demand.

**Fig. 1:**
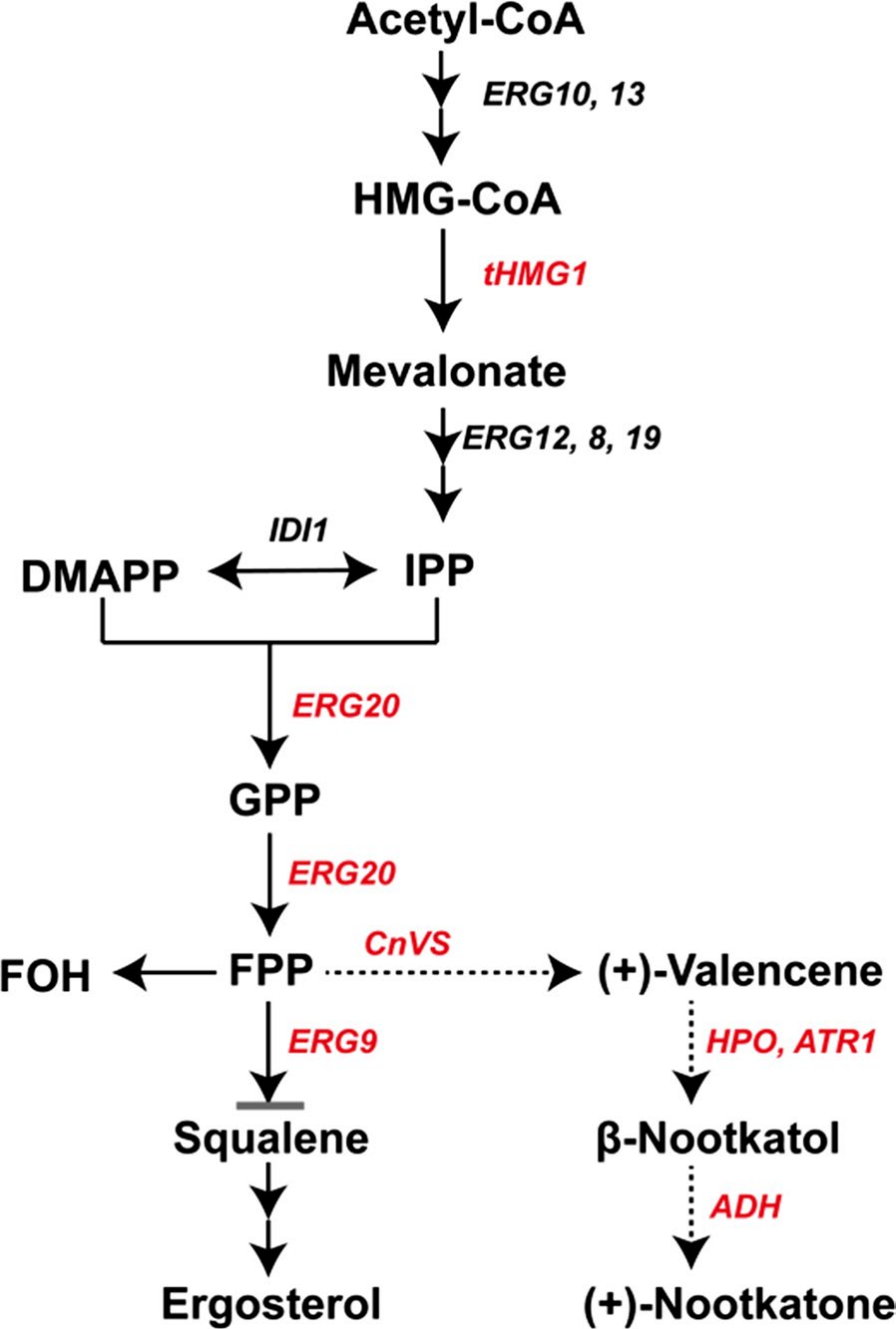
Scheme of (+)-nootkatone biosynthesis based on the MVA pathway in *S. cerevisiae.* To increase the metabolic flux of the MVA pathway in *S. cerevisiae*, tHMG1 was overexpressed and the competitive ERG9 pathway was dynamically controlled by replacing its endogenous promoter with P_*HXT1*_. ERG20 was simultaneously fused with CnVS of *C. nootkatensis* and overexpressed to channel the metabolic flux to (+)-valencene production. The production of (+)-nootkatone was achieved by the overexpression of HPO, ATR1, and the indicated dehydrogenases

Although (+)-nootkatone production in microbial cell factories has been attempted, the yields are unacceptably low and development of an economically viable bioprocess for (+)-nootkatone production still remains challenging. In this study, we constructed the nootaktone biosynthesis pathway in *S. cerevisiae* by overexpressing the (+)-valencene synthase CnVS and the P450 enzyme HPO in combination with engineering the endogenous MVA pathway (Fig. 1). Importantly, several dehydrogenases were screened for catalyzing the conversion of β-nootkatol to (+)-nootkatone. The yield of (+)-nootkatone reached 59.78 mg/L in our constructed *S. cerevisiae* cell factory, thus providing a solid basis for high-level production of (+)-nootkatone.

## Results

### Production of (+)-valencene by CnVS expression in *S. cerevisiae*

**Fig. 2:**
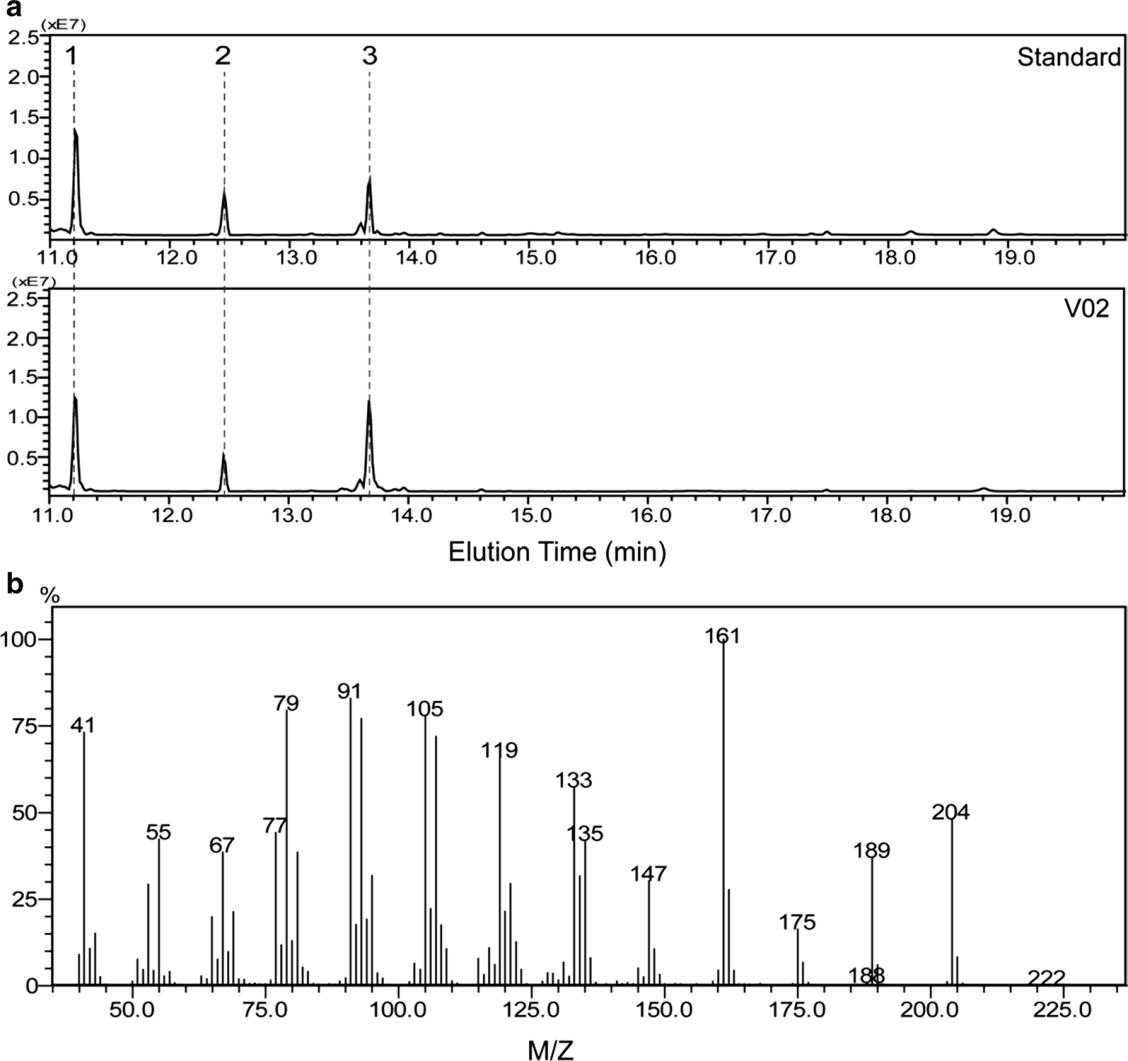
GC–MS analysis of the extracted n-dodecane phase of shake-flask cultures of strain V02. **a** The GC–MS profiles of products produced by V02 strain and (+)-valencene standard. **b** Mass spectrum of (+)-valencene (peak 3). Peak 1 and peak 2 were identified as n-tridecane and n-tetradecane, which could be the contaminants from the used dissolvent n-dodecane. The GC–MS profiles of solvent control and the obtained MS–MS profiles of n-tridecane and n-tetradecane were included in the Additional file 1: Fig. S2

*Saccharomyces cerevisiae* has been proven to be an ideal host for the production of highly-valued terpenoid compounds by leveraging its inherent capacity to synthesize terpenoid precursors through the MVA pathway [1, 2]. As (+)-valencene is the precursor for (+)-nootkatone biosynthesis, we first explored the biosynthesis of (+)-valencene in *S. cerevisiae* W303 by expressing the robust (+)-valencene synthase CnVS (strain V02). n-Dodecane (10%, v/v) was added to the fermentation medium for in situ separation of the produced (+)-valencene given the low solubility of (+)-valencene in the aqueous phase and its high volatility [15]. Our growth test revealed that 10% n-dodecane showed only a marginal effect while (+)-valencene up to 500 mg/ml elicited hardly any inhibitory effect on the yeast growth (Additional file 1: Fig. S1). GC–MS analysis of the n-dodecane phase procured from the V02 cultivation revealed (+)-valencene as the dominant product, reaching about 11.6 mg/L (Fig. 2). The mass spectrum of the produced (+)-valencene was in agreement with that of the authentic standard.

### Engineering the MVA pathway to improve (+)-valencene production

**Fig. 3:**
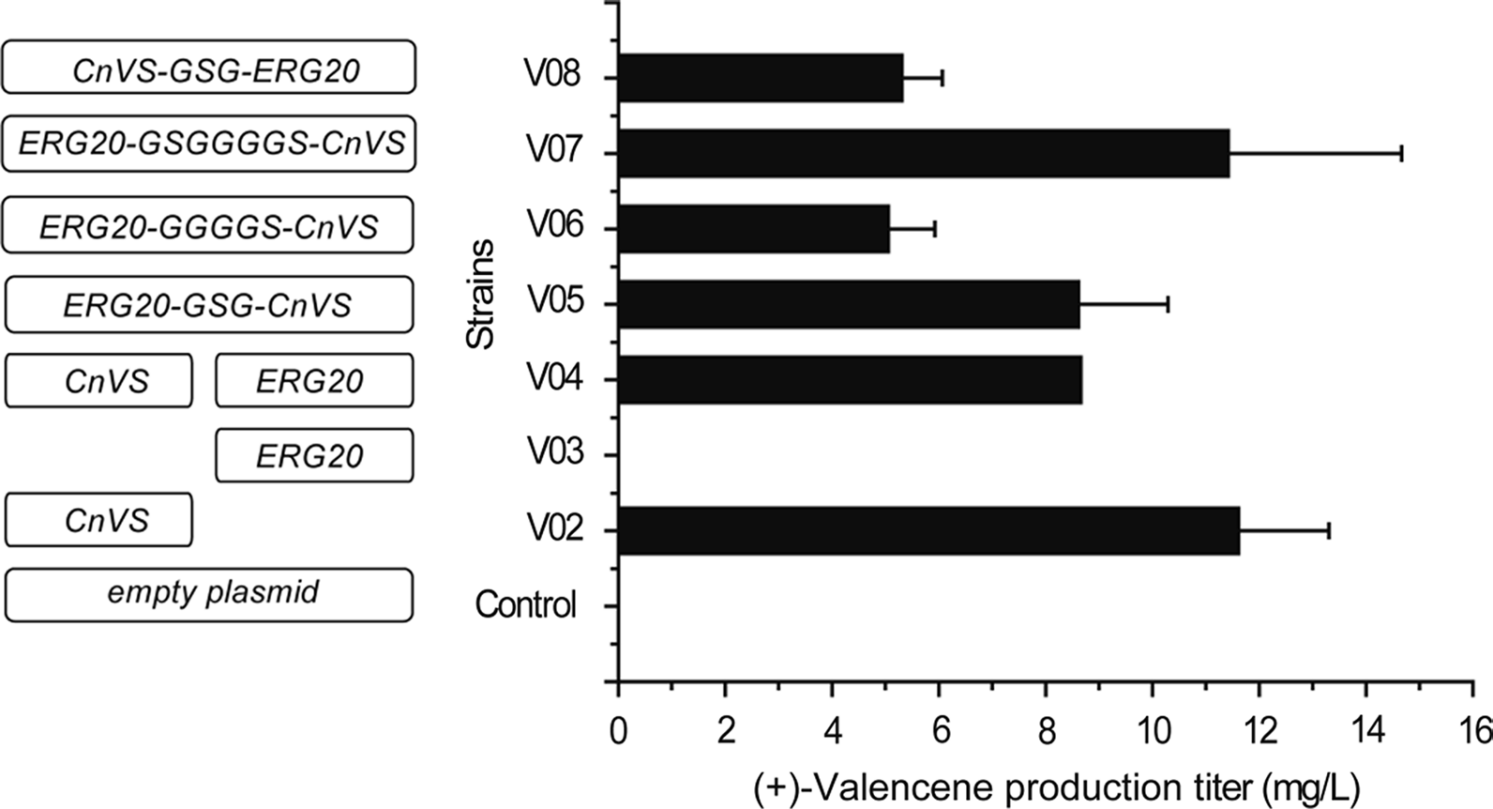
(+)-Valencene production in *S. cerevisiae* strains overexpressing CnVS and ERG20 or their different fused forms. The overexpressed proteins are shown accordingly

To further improve (+)-valencene production, FPP synthase ERG20 that catalyzes the condensation of IPP and its isomer DMAPP into GPP and consecutively the incorporation of GPP with one extra IPP to form FPP [1], was first overexpressed in V04 to increase the metabolic flux to FPP. Considering that FPP is also the substrate for squalene and farnesol synthesis, we tested the effect of expressing the fused form of ERG20 with CnVS using three different flexible linkers, namely GSG, GGGGS, and GSGGGGS, on (+)-valencene formation with an expectation to minimize the competitive FPP consumption by channeling the metabolic flux to the target product [24–26]. Overall, the results revealed that neither the ERG20 overexpression alone nor its fused expression with CnVS improved (+)-valencene yield (Fig. 3), which was probably due to a short supply of precursors. Specifically, strains V06 and V08 expressing ERG20-GGGGS-CnVS and CnVS-GSG-ERG20 expression, respectively, even produced a significantly lower amount of (+)-valencene than V02 and strains with other fused forms.

**Fig. 4:**
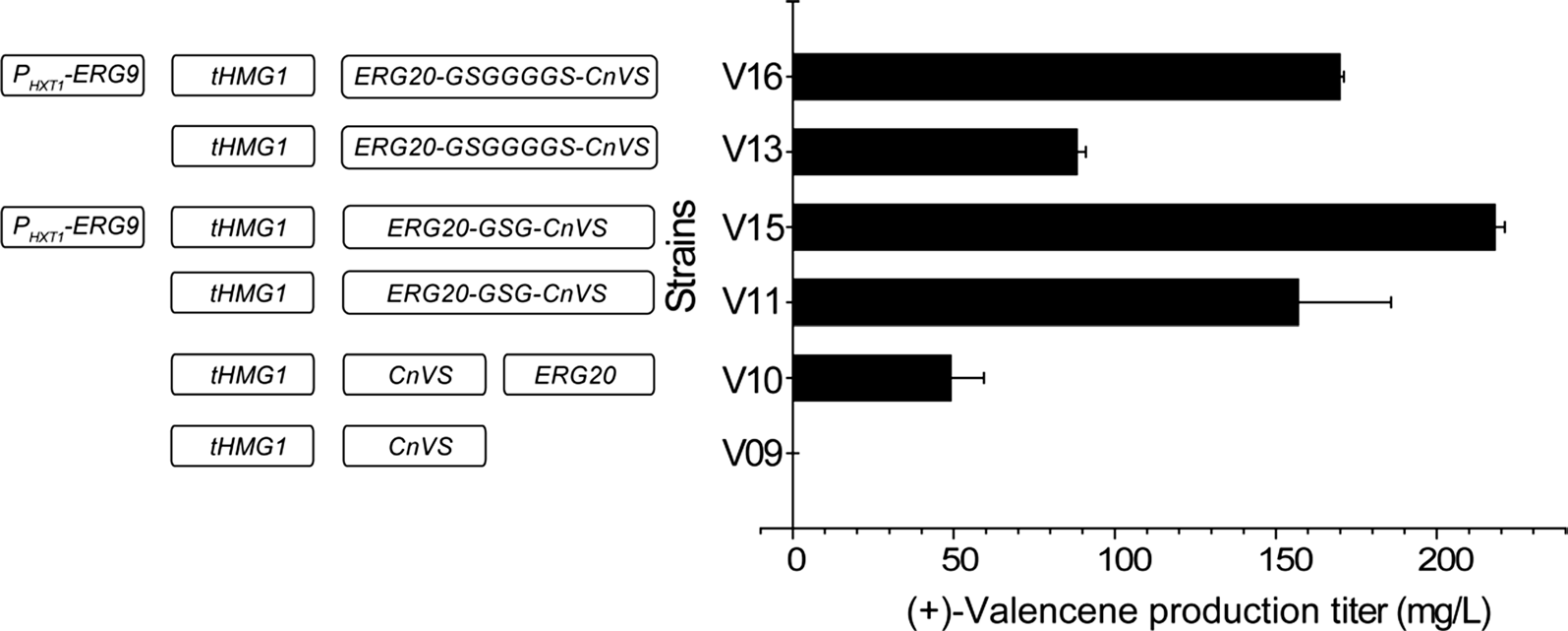
The effects of tHMG1 overexpression and dynamic downregulation of ERG9 on the production of (+)-valencene in different metabolically engineered *S. cerevisiae* strains. tHMG1 was overexpressed in strains V02, V04, V05, and V06, resulting in strains V09, V10, V11, and V13, respectively. The production of (+)-valencene was determined by GC-FID analysis. The ERG9 was further downregulated in strain V11 and V13 by replacing its endogenous promoter with *P*_*HXT1*_ promoter, resulting in V15 and V16 strains, respectively. The production of (+)-valencene was determined by GC-FID analysis

Multiple studies have shown that overexpression of the catalytic domain of the rate-limiting step enzyme tHMG1 has a profound effect on the metabolic flux of the MVA pathway [27]. This truncated HMG1 has also been reported to be capable of relieving the negative feedback inhibition of the MVA pathway [27]. We thus overexpressed *S. cerevisiae* tHMG1 to increase the precursor supply for (+)-valencene synthesis. The results showed that tHMG1 overexpression greatly enhanced the production of (+)-valencene (comparing V10, V11, and V13 to V04, V05, and V07, resepectively) (Figs. 3, 4). Specifically, valencence level in V10 was almost sixfold higher than that in V04, reaching about 48.99 mg/L of cell culture. Of note, when combined with the fused overexpression of ERG20 and CnVS (V05 and V07), tHMG1 overexpression boosted (+)-valencene production by 9- and 14-fold to 88.20 mg/L (V13) and 156.95 mg/L (V11), respectively, compared with their corresponding parental strains, manifesting the effects of fusing ERG20 with CnVS. In the V09 strain, tHMG1 was overexpressed to enhance the precursor supply without ERG20 overexpression. However, the growth of V09 was severely compromised, which resulted in hardly any production of (+)-valencene. The growth inhibition may be attributed to the accumulation of IPP and DMAPP, which has been proven to be toxic to the cell [28, 29]. Comparing the growth and (+)-valencene production of the V09 and V10 strains suggested that overexpressing the downstream *erg*20 gene relieved the growth inhibition of *S. cerevisiae*, reflecting a beneficial effect of ERG20 overexpression on the simultaneous tHMG1 overexpression. Altogether, these results indicate that more FPP would be channeled to CnVS when it is appropriately fused with ERG20 on condition that there are sufficient IPP and DMAPP precursors.

FPP is a key branch point in the *S. cerevisiae* MVA pathway. The majority of FPP flux is directed to squalene synthase ERG9, which is dedicated to the synthesis of ergosterol [14, 30]. Dynamically down-regulating the ERG9 competition pathway has thus been proven very effective in enhancing the production of sesquiterpene compounds [14, 30]. Biochemical analysis has demonstrated that the turnover rate of ERG9 is 1000-fold higher than CnVS [31], implying that down-regulating the ERG9 competitive pathway would contribute to (+)-valencene biosynthesis. In addition, FPP can also be converted to farnesol by diphosphatase-catalyzed hydrolysis [32]. We thus replaced the *erg9* promoter with the glucose response *HXT1* promoter,which displays low transcription levels at low glucose concentrations [33], expecting to reduce the FPP consumption by the ergosterol pathway at the late stage of cell growth. As shown in Fig. 4, ERG9 down-regulation further increased (+)-valencene production in strains V11 and V13, and the highest yield was achieved in V15 with a 19-fold increase compared to V02 to 217.95 mg/L. To check whether farnesol formation by endogenous diphosphatases competes for the FPP pool, farnesol was determined in the constructed (+)-valencene producing strain. The results showed that significant farnesol accumulation was not observed in all tested strains (Additional file 1: Fig. S3), indicating that the catalytic efficiency of CnVS is much higher than diphosphatases and elimination of diphosphatases is not required in the present study.

### Overexpression of (+)-valencene oxidase for the biosynthesis of (+)-nootkatone

**Fig. 5:**
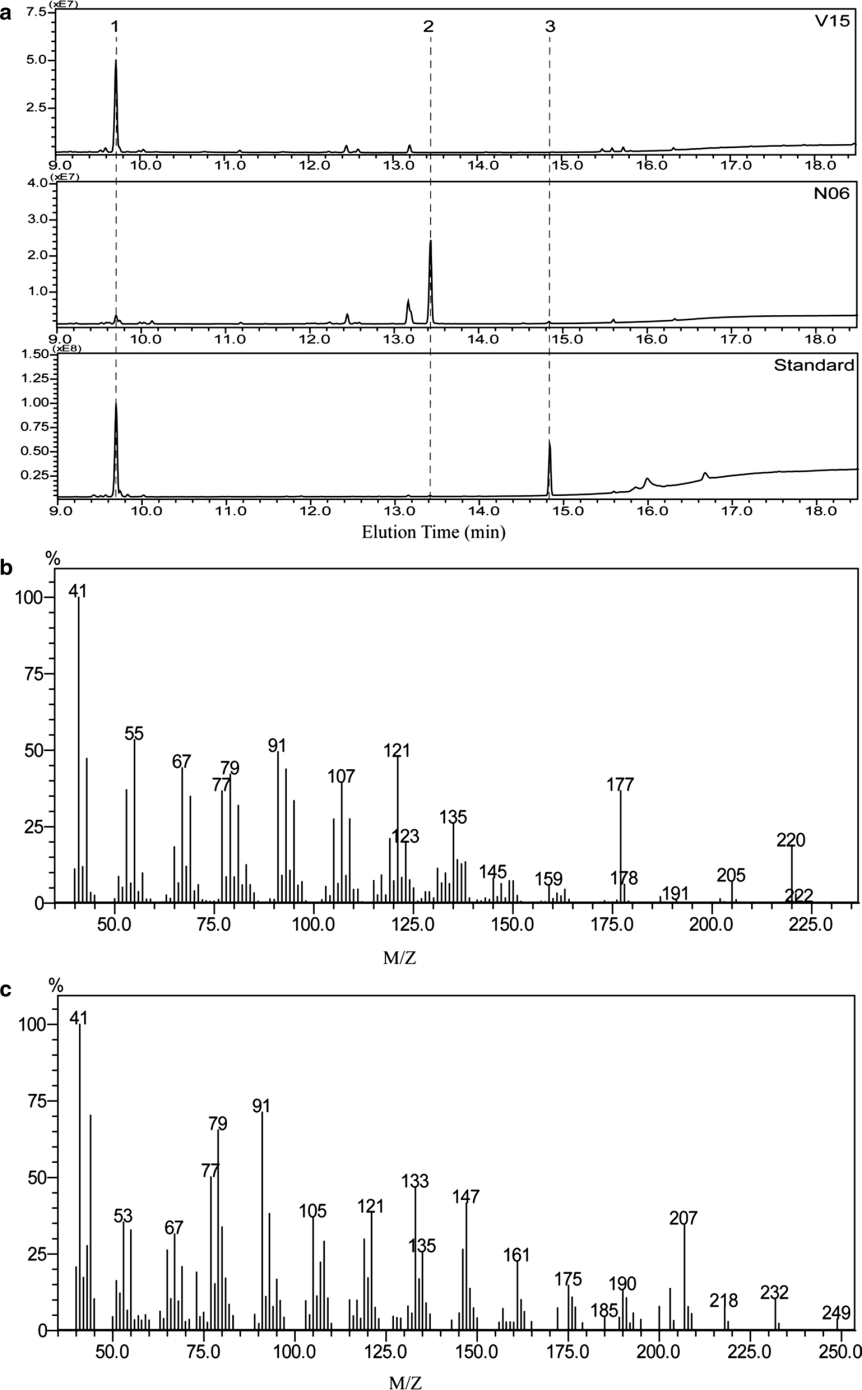
Production of (+)-nootkatone by HPO and ATR1 overexpression in strain V15. GC-MS profiles (**a**) of products produced by strain V15, strain N06 and standard mixture of (+)-valencene and (+)-nootkatone are presented. The MS spectra of peak 2 (**b**) and 3 (**c**) are shown and fitted to the standard spectra of β-nootkatol and (+)-nootkatone, respectively

Allylic oxidation of (+)-valencene by (+)-valencene oxidase provides an attractive route for the production of (+)-nootkatone [4]. Reported (+)-valencene oxidases include P450 enzymes and lipoxygenase [10, 21]. P450 enzymes primarily oxidize (+)-valencene to β-nootkatol while lipoxygenases produce a significant amount of (+)-nootkatone [10, 21]. We overexpressed the P450 HPO mutant V482I/A484I and lipoxygenase ValOx from *P. sapidus*, respectively, in the (+)-valencene production strain V15. GC analysis demonstrated that the amount of (+)-valencene decreased dramatically in V15 overexpressing HPO V482/A484I (strain N06) while only minor amounts of (+)-nootkatone as well as a significant amount of β-nootkatol were identified in the product profiles (Fig. 5). Their MS profiles were in agreement with the reported standard profiles (Fig. 5b, c) [10]. The yield of (+)-nootkatone was determined to be 9.66 mg/L whereas quantification of nootakatol was not performed due to the lack of a standard. Lipoxygenase ValOx overexpression in V15 did not produce any oxidized product (Additional file 1: Fig, S4).

(+)-Valencene is a volatile compound with very low solubility. n-Dodecane was usually over-layered on the culture for in situ separation to facilitate the quantification of valencene. However, (+)-valencene would be quickly extracted by the n-dodecane once it is produced, making the concentration of intracellular (+)-valencene too low to be efficiently transformed further. To increase (+)-valencene solubility in aqueous solution and improve its oxidation by HPO, two different solvents (DMSO and Triton X-100) were examined for their effects on *S. cerevisiae* growth and the production of (+)-nootkatone (Additional file 1: Figs. S5 and S6). Increasing DMSO concentrations hardly affect cell growth until it reached 8% (v/v) (Additional file 1: Fig. S5). The highest yield of (+)-nootkatone was obtained at 1% DMSO (Additional file 1: Fig. S5). Although the cell growth were similar to DMSO when increased concentrations of Triton X-100 (0–0.1%) were applied (Additional file 1: Fig. S6), the production of (+)-nootkatone was only slightly increased. Therefore, 1% DMSO was chosen for all the subsequent fermentation experiments.

### Screening dehydrogenases for improvement of (+)-nootkatone production

Having found that β-nootkatol was produced as the predominant product in the HPO V482/A484I overexpression strain (N06), we next tried to find a solution to improve the oxidation of β-nootkatol to (+)-nootkatone, not only because this conversion may constitute the bottleneck for (+)-nootkatone production, but also due to the fact that β-nootkatol accumulation is toxic to the cell [10]. In the analysis of the oxidized products from (+)-valencene by P450 enzymes, several studies have noticed that the oxidation of β-nootkatol to (+)-nootkatone is probably not catalyzed by P450 enzymes but instead by some putative dehydrogenases in the production host [10, 18]. Specifically, efficient oxidation of β-nootkatol to (+)-nootkatone has been reported by overexpressing an endogenous dehydrogenase ADH-C3 in *Pichia pastoris* [23]. Several dehydrogenases from *S. cerevisiae*, *P. pastoris,* and plants were thus tested for their activity in converting β-nootkatol to (+)-nootkatone in the present study.

There are seven alcohol dehydrogenases (ADH1-7) in *S. cerevisiae* [34]. Among them, ADH2 is reported to be able to convert ethanol to acetaldehyde while ADH6 and ADH7 display broad substrate specificity. ADH-C3 of *P. pastoris* is the only dehydrogenase thus far reported to catalyze the oxidation of β-nootkatol to (+)-nootkatone [23], which shares 37% and 34% sequence identity with ADH6 and ADH7, respectively. Since (+)-nootkatone is naturally produced by various plants including *C. sinensis,* one short-chain dehydrogenase/reductase superfamily (SDR) dehydrogenase ABA2 with reported hydroxylation activity arouse our interest in view of its broad substrate specificity [35]. Another SDR family dehydrogenase ZSD1 from *Zingiber zerumbet* has been reported to oxidize the hydroxyl group of monoterpenoid and sesquiterpenoid to keto group [36]. Therefore, together with ADH2 and ADH6 of *S. cerevisiae*, and ADH-C3 of *P. pastoris*, ABA2 and ZSD1 were chosen for overexpression in the N06 strain.

**Fig. 6:**
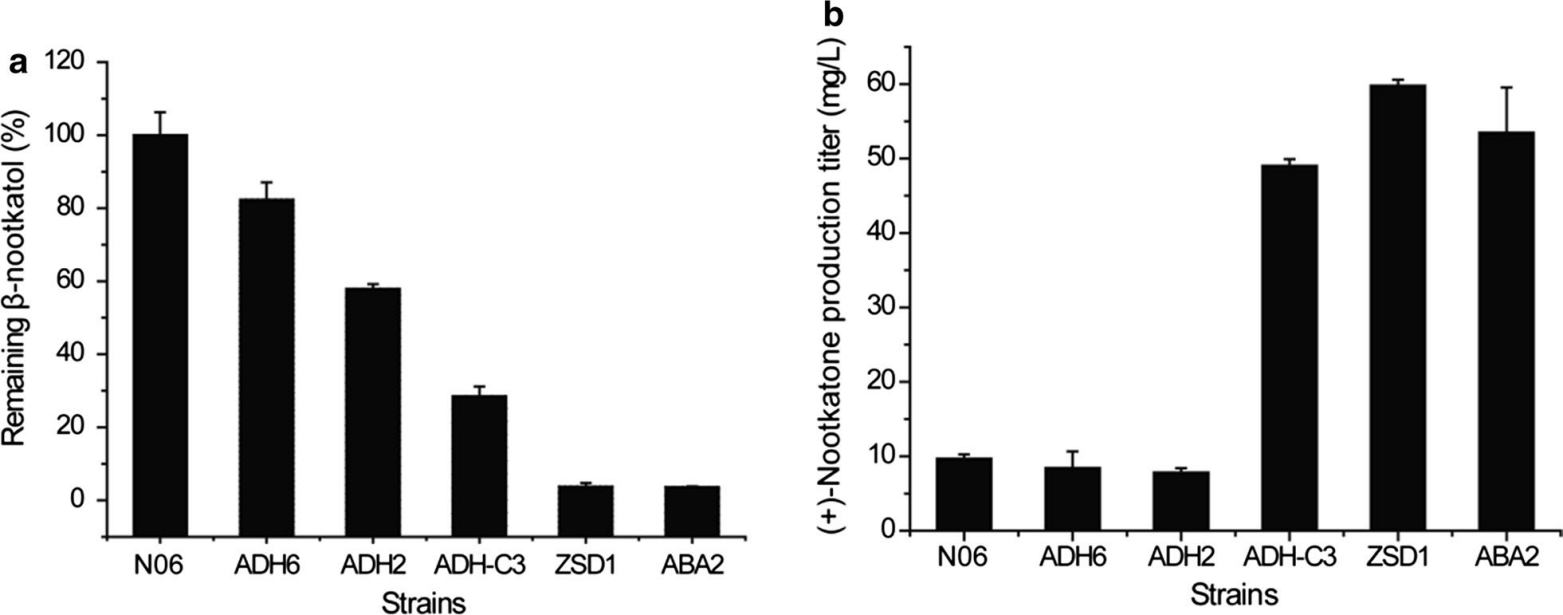
The effects of dehydrogenase overexpression on the production of β-nootkatol (**a**) and (+)-nootkatone (**b**). Various dehydrogenases (ADH6, ADH2, ADH-C3, ZSD1, and ABA2) were overexpressed in strain N06. The production of β-nootkatol and (+)-nootkatone in these strains were determined by GC-FID and compared to that of N06. Due to the lack of β-nootkatol standard, the amount of β-nootkatol in dehydrogenase overexpression strains was shown as the relative percentage to that of N06 strain

As seen in Fig. 6, consistent with previous results, ADH-C3 was capable of conferring efficient (+)-nootkatone production with an yield of 49.01 mg/L. Notably, ZSD1 or ABA2 expression also significantly increased the yield of (+)-nootkatone to 59.78 mg/L and 53.48 mg/L, respectively, with a decrease in the amount of β-nootkatol (Fig. 6). In contrast with ADH-C3, ZSD1, and ABA2, there was no significant change in the yield of (+)-nootkatone in ADH2- and ADH6-overexpressing strains compared with N06 although the yield of β-nootkatol slightly decreased. Overexpression of these two dehydrogenases may thus interfere with the expression of CnVS or HPO in the resultant production strains.

## Discussion

(+)-Nootkatone is a highly valued oxidized sesquiterpene compound, displaying a variety of biological activities and desirable properties for use as aromatics, pharmaceuticals, and biofuels [4]. Efficient synthesis of (+)-nootkatone via biotechnological approaches is thus urgently needed. *S. cerevisiae* is considered as the generally regarded as safe organism and possesses endogenous MVA pathway for the synthesis of terpenoid precursors, i.e., GPP for monoterpene and FPP for sesquiterpene [1, 2]. In addition, *S. cerevisiae* is a suitable eukaryotic host for the efficient expression of heterologous plant terpene biosynthetic enzymes such as terpene synthase and P450 enzymes [1, 2]. However, the biosynthesis of (+)-nootkatone in *S. cerevisiae* has not been explored systematically.

Sufficient supply of the (+)-nootkatone precursor, (+)-valencene, represents the first challenge needed to be addressed for the efficient de novo synthesis of (+)-nootkatone. Among others, the activity of the (+)-valencene synthase is the key for the effective synthesis of (+)-valencene [11–13]. Compared to other sesquiterpene synthases, activities of the identified (+)-valencene synthases are usually 3–5 times lower [11–13]. Some of these (+)-valencene synthases display poor product specificity, producing significant amounts of various by-products [12]. CnVS from *C. nootkatensis* was reported to be more selective and robust, displaying stronger tolerance to different pH and temperatures [13]. In the present study, CnVS overexpression alone in *S. cerevisiae* produced 11.61 mg/L (+)-valencene (Fig. 2). This titer, although was already higher than most of the reported literature, is still far from being sufficient to scale up for commercial applications. Further increasing the activity of (+)-valencene synthase by enzyme engineering thus holds great potential to boost the production of (+)-valencene. Built upon this, another limiting factor for (+)-valencene production is the requirement of an ample supply of the precursor FPP. Numerous studies have explored various approaches for reallocating the metabolic flux in *S. cerevisiae* to the production of the sesquiterpenes [1, 2]. Among them, overexpression of the sesquiterpene synthase fused with ERG20 [26], overexpression of the rate-limiting enzyme tHMG1 [30], and dynamic down-regulation of the competitive ERG9 pathway [14], have proven to be powerful for fortifying the flow of intracellular FPP pool for product formation. In the present study, the combination of these approaches increased the production of (+)-valencene to 217.95 mg/L (Fig. 4). Notably, the production of farnesol remained consistently low in the production strains, implicating that FPP supply is still limiting for the production of (+)-valencene. Other approaches including overexpression of the global regulator UPC2-1 of the MVA pathway and isopentenyl pyrophosphate (IPP) isomerase IDI1 [24], and even overexpression of all the MVA pathway enzymes [37], could be employed for increasing (+)-valencene production. Since (+)-valencene exerts strong citrus fruit odor itself, it is of interest to the food and cosmetic industry on its own. Thus, metabolically engineered *S. cerevisiae* in our present study represents a valuable microbial cell factory for high-level production of (+)-valencene.

The efficient oxidation of (+)-valencene to (+)-nootkatone constitutes the second challenge to be addressed for de novo synthesis of (+)-nootkatone from simple carbon sources in microbial cell factories. Whole-cell system or crude protein extracts from different sources, i.e. plant, bacteria, and fungus have been screened for the biotransformation of (+)-valencene to (+)-nootkatone [10, 17–19, 38, 39]. The regioselective oxidation of allylic group of (+)-valencene has been achieved using P450 enzymes by two successive oxidation steps: first to β-nootkatol followed by second oxidation to (+)-nootkatone. However, most of the reported P450 enzymes (CYP71AV8, CYP71D51v2, and HPO) are found to be more efficient in the oxidation of (+)-valencene to β-nootkatol while the efficiency of further conversion of β-nootkatol to (+)-nootkatone is extremely low (Fig. 5) [10, 17–19, 38]. This seriously impedes the efficient biosynthesis of (+)-nootkatone. The P450 enzyme HPO from *H. muticus* has been biochemically characterized to catalyze the allylic oxidation of various sesquiterpenoid compounds [19]. With (+)-valencene as substrate, however, HPO predominately converts it to β-nootkatol. Interestingly, HPO was capable of catalyzing the successive oxidation of allylic group of premnaspirodiene to produce solavetivol and solavetivone [19]. Although the first oxidation reaction producing solavetivol was kinetically favored with threefold higher catalytic efficiency than the second oxidation reaction to solavetivone [19], the capability of HPO to oxidize solavetivol is much higher than β-nootkatol. Notably, our bioinformatics analysis (Additional file 1: Fig. S7) implicated that, although the calculated binding free energy of solavetivol (− 25.1 ± 2.5 kcal/mol) is lower than that of β-nootkatol (− 22.7 ± 3.7 kcal/mol), this difference may not be enough to account for the high efficiency catalysis of solavetivol, which is somehow able to establish more favorable interactions with HPO than β-nootkatol. Mutations targeting the substrate recognition site have been performed to investigate their influence on the substrate specificity. Although the V482I/A484I HPO mutant showed fivefold higher catalytic efficiency in oxidizing (+)-valencene to β-nootkatol compared to wild-type HPO, its catalytic efficiency in (+)-nootkatone synthesis was not improved [19], which was also shown in our present study. It thus seems that it is inherently difficult for P450 enzymes to catalyze the second oxidation reaction. Besides P450, the lipoxygenase ValOx of *P. sapidus* has been also reported to predominantly oxidize (+)-valencene into (+)-nootkatone [21, 22]. In our study, no oxidation products of (+)-valencene was identified when ValOx was overexpressed in the (+)-valencene production strain V15. We further examined whether ValOx was expressed in the recombinant *S. cerevisiae* strain by fusing GFP to the C-terminal end of ValOx. A clear green fluorescence was observed in the ValOx-GFP overexpression strain (Additional file 1: Fig. S8), suggesting that ValOx-GFP was indeed expressed. It remains unclear why the overexpressed ValOx is not functional in the (+)-valencene production *S. cerevisiae* strain. Other possibilities are that the proper folding and/or subcellular localization of ValOx are not favourable in the constructed strain considering that expression of enzymes of fungal origin in *S. cerevisiae* often encounter difficulties.

While exploring the P450 enzymes for the oxidation of (+)-valencene to (+)-nootkatone, several studies have noted that the generated limited amount of (+)-nootkatone does not originate from the P450 enzyme activities but instead from some endogenous dehydrogenases of host cells [10, 18]. Specifically, Wriessnegger et al. reported that the dehydrogenase ADH-C3 of *P. pastoris* was capable of oxidizing β-nootkatol to (+)-nootkatone [23]. The coupling between specific P450 enzymes and dehydrogenases may therefore be a promising alternative strategy for the efficient oxidation of (+)-valencene to (+)-nootkatone. In addition, this combined enzymatic route has a distinct advantage of regenerating cofactors NADPH consumed by P450 enzymes, thus relieving the potential oxidative stress resultant from P450 catalysis in host cells. However, this strategy has not been explored extensively. In our present study, besides ADH-C3 of *P. pastoris*, we also found that the SDR family dehydrogenases including ABA2 of *C. sinensis* and ZSD1 of *Z. zerumbet* were capable of efficiently converting β-nootkatol into (+)-nootkatone. Overexpression of ADH-C3, ABA2, and ZSD1, respectively, significantly promoted the conversion of β-nootkatol (Fig. 6) with (+)-nootkatone production in the ZSD1 overexpression strain being increased by 20% compared to that of ADH-C3. Both ZSD1 and ABA2 belong to plant SDR superfamily enzymes, which are the most important terpene modifying enzymes [35, 36]. These SDRs are generally NAD(P)-dependent enzymes involved in secondary metabolism and display wide and promiscuous substrate specificity in catalyzing the oxidation of alcohol, sterol, saccharides, and aromatic compounds [40]. While ZSD1 has been biochemically characterized to catalyze the oxidation of sesquiterpene alcohol 8-hydroxy-α-humulene and monoterpene alcohol borneol in a NAD-dependent manner [36], ABA2 has been reported to be involved in the sesquiterpenoid ABA synthesis and to catalyze the oxidation of xanthoxin to abscisic aldehyde [35]. Similar to our present study, two SDRs from peppermint and spearmint and one from *Artemisia annua* have been found to be involved in the biosynthesis of monoterpene [41, 42]. Together with our results, plant SDRs are thus shown to be important terpene modifying enzymes and will serve as new tools for the biosynthesis of terpenoids. Previous work speculated that a dehydrogenase might be present in *S. cerevisiae* for the oxidation of β-nootkatol to (+)-nootkatone [10, 18]. However, ADH2 and ADH6 from *S. cerevisiae* failed to convert β-nootkatol to (+)-nootkatone, suggesting that other unidentified dehydrogenases might be responsible for this activity. The remaining *S. cereviasiae* ADHs should be tested to see whether there indeed exists an orthologous ADH being able to convert nootkatol to (+)-nootkatone. Nevertheless, our present study identified two effective dehydrogenases from plants, which will serve as valuable tools in the construction of (+)-nootkatone microbial cell factories.

Although the production of (+)-nootkatone reached 852.30 mg/L using *Yarrowia lipolytica* as whole cell biocatalysts for the oxidation of in vitro fed (+)-valencene [38], the (+)-nootkatone production using the in situ produced (+)-valencene in different microorganisms remained relatively low (Table 1). The constructed (+)-nootkatone producing *S. cereviase* strain in the present study can be used as a suitable platform to further improve nookatone biosynthesis. Upon expression of the two identified plant-derived ADHs, the residue amount of β-nootkatol was proven to be relatively low, suggesting that the main bottleneck for the production of (+)-nootkatone is not the oxidation of β-nootkatol anymore in the present stage. The limiting factors for further improvement in (+)-nootkatone production are probably the MVA pathway precursor supply and/or the catalytic efficiency of (+)-valencene synthase. Other established approaches for MVA pathway engineering, including overexpression of the MVA pathway global regulator UPC2-1 and IPP isomerase IDI1, can be utilized to increase the precursor supply [24, 37]. More effective (+)-valencene synthase can be identified by either database mining or direct evolution of CnVS.

**Table 1:** (+)-Nootkatone production in different microorganisms using various engineering approaches

Microorganisms	Strategies	Titer	References
*E. coli*	Using recombinant *E. coli* expressing CYP109B1 from *B. subtilis* as whole cell biocatalyst for the oxidation of in vitro fed (+)-valencene	3.32 mg/L	[17]
*S. cerevisiae*	Using yeast WAT11 expressing CYP71D51v2 from *N. tabacum* as whole cell biocatalyst for the oxidation of in vitro fed (+)-valencene	3.00 mg/L	[10]
*Chaetomium globosum*	Using submerged cultures of *C. globosum* as whole cell biocatalyst for the oxidation of in vitro fed (+)-valencene	25.00 mg/L	[39]
*Yarrowia lipolytica*	Using *Y. lipolytica* as the whole cell biocatalyst for the oxidation of (+)-valencene (3.3 g/L) in the fed orange essential oil in a three phase partitioning bioreactor	852.30 mg/L	[38]
*S. cerevisiae*	Co-expression of (+)-valencene synthase Cstps1 with a chicory cytochrome P450 mono-oxygenase CYP71AV8	0.04 mg/L	[18]
*S. cerevisiae*	Co-expression of (+)-valencene synthase CnVS with a (+)-valencene oxidase CYP706M1 from *C. nootkatensis*	0.144 mg/L	[20]
*P. pastoris*	Co-expression of CnVS, HPO and ADH-C-3 in combination with overexpression tHMG1 in *P. pastoris*	35.00 mg/L (flask fermentation) 208.00 mg/L (fed-batch fermentation)	[23]
*S. cerevisiae*	Combining CnVS overexpression with various MVA pathway engineering approaches including the expression of CnVS and ERG20 as fused proteins, overexpression of tHMG1, and downregulating the ERG9 competitive pathway; achieve (+)-valencene oxidation by simultaneous overexpression of HPO and dehydrogenases ZSD1	59.78 mg/L	The present study

The catalytic efficiency of HPO could be another limiting factor for the efficient (+)-nootkatone biosynthesis. Generalizable strategies that support the functional expression of plant cytochrome P450 enzymes for biosynthesis pathway optimization in *S. cerevisiae* have been developed [43]. Considering that the appropriate coupling between P450 enzymes and CPRs including their relative expression is critical for the overall catalytic efficiency [44], the performance of HPO could be further enhanced by using promoters with different strengths to optimize the relative expression level of HPO and ATR1. On the other hand, it is generally accepted that the optimal redox partners for a P450 enzyme should be homogeneous ones [45]. In this regard, the CPR from *H. muticus* specific for HPO should be identified and employed with the possibility of improving HPO catalytic efficiency. Moreover, additional expression of cytochrome b5 has been also reported to be beneficial for the performance of P450 enzymes [37], which merits test in the future.

## Conclusion

In conclusion, our present study first addressed the challenge of de novo (+)-valencene production by combining CnVS overexpression with various MVA pathway engineering approaches including the expression of CnVS and ERG20 as fused proteins, overexpression of tHMG1, and downregulating the ERG9 competitive pathway. Overall these efforts led to significantly improved production of (+)-valencene with a yield of 217.95 mg/L. Secondly, we tackled the (+)-valencene oxidation by simultaneous overexpression of HPO and dehydrogenases ABA2 and ZSD1. Altogether the production of (+)-nootkatone reached 59.78 mg/L. The constructed (+)-nootkatone biosynthesis *S. cereviase* strain provided a suitable platform for further development of a viable bioprocess for the industrial production of (+)-nootkatone.

## Methods

### Strains, media and growth conditions

*Escherichia coli* DH5α used for cloning purposes was routinely cultured in Luria–Bertani medium with the appropriate amount of antibiotics (100 μg/ml ampicillin or 40 μg/ml kanamycin) at 37 °C. *S. cerevisiae* W303-1A (*MATa, LEU2*-*3,112 TRP1*-*1 CAN*-*100 URA3*-*1 ADE2*-*1 HIS3*-*11,15*) was used as the parent strain for all yeast strain constructions. All yeast strains were cultivated in either yeast extract-peptone-dextrose medium (YPD, 1% yeast extract, 2% peptone, and 2% glucose) or yeast nitrogen base medium [YNB, yeast nitrogen base 0.17%, 0.13% CM, (NH_4_)_2_SO_4_ 0.5%, 2% glucose]. YNB medium dropping out certain components (leucine, histidine, tryptophan, and uracil) or adding geneticin (G418, 400 mg/L) were used for auxotroph or geneticin resistance selection of *S. cerevisiae* transformants. The solid YPD and YNB media were prepared by adding 20 g/L agar into the liquid media.

### Plasmid and strain construction

Phusions High Fidelity DNA polymerase (Thermo Fisher Scientific, Germany) was used for gene amplification according to the recommended protocol. Ligations in the present study were performed with T4 DNA ligase or T5 exonuclease-dependent assembly [46]. The DNA sequence of *C. nootkatensis* CnVS (Genbank accession: JC245925.1) was codon optimized and synthesized (Tsingke, Qingdao, China). The *P*_*GAL1*_ promoter, the *CnVS* gene, and the *CYC1* terminator were orderly inserted in the pRS305 plasmid at *Sma*I and *Sac*I sites to generate the pRS305-CnVS plasmid. The *ERG20* and *CnVS* fusion gene was constructed by inserting the GSG, GGGGS, or GSGGGGS linker between these two genes with nested PCR. The *P*_*GAL1*_ promoter, the ERG20 and *CnVS* coding sequences, and the *CYC1* terminator were assembled by T5 exonuclease-dependent assembly, and inserted at *Xho*I and *Sac*I sites of pRS305 to obtain the pRS305-E3C, pRS305-C3E, pRS305-E5C, pRS305-E7C plasmid, respectively. tHMG1 was amplified from the genomic DNA of *S. cerevisiae* W303-1A and assembled between the P_*TPI1*_ promoter and the T_*CYC1*_ terminator, which was then inserted between BamHI and XhoI sites of pRS306 to obtain the pRS306-tHMG1 plasmid. To replace the endogenous promoter of *ERG9* with the P_*HTX1*_ promoter, an integration cassette was constructed by assembling the upstream homologous region of the P_*ERG9*_ promoter (70 bp), loxP-*kanMX*-loxP, P_*HTX1*_, and the downstream homologous region of P_*ERG9*_ promoter (70 bp) in the desired order. The *H. muticus* HPO (EF569601.1) mutant (V482I A484I) gene and the *A. thaliana atr1* (NM_118585.3) were codon optimized and synthesized by Tsingke. Their expression cassette were then separately constructed under the control of the P_*GAL1*_ promoter and the T_*CYC1*_ terminator by inserting the corresponding sequences adjacently in pRS304 between *Eco*53KI and *Spe*I sites to obtain the pRS304-HPO-ATR1 plasmid. Similarly, the codon optimized *ValOx* gene (HF913621.1) of *P. sapidus* was assembled between the P_*GAL1*_ promoter and the T_*CYC1*_ terminator, and the resulted expression cassette was inserted in pRS304 between *Eco53*KI and SpeI sites to obtain the pRS304-ValOx plasmid. The coding sequences for dehydrogenases ADH2 (Genbank: NM_001182812.1) and ADH6 (NM_001182831.3) of *S. cerevisiae*, ADH-C3 (XM_002492172) of *P. pastoris*, ZSD1 (AB480831.1) of *Z. zerumbet*, and ABA2 (HM036684.1) of *C. sinensis* were codon optimized and overexpressed under the control of the P_*GAL1*_ promoter and T_*CYC1*_ terminator, which were inserted between EcoRI and SacI sites in pRS423, respectively. All the plasmids constructed were verified by DNA sequencing (Tsingke, Qingdao, China). The strains and the corresponding plasmids used for their construction are listed in Table 2.

**Table 2:** Strains used in this study

Strains	Description	Source
*S. cerevisiae* W303-1A	*MATa (LEU2*-*3,112 TRP1*-*1 CAN*-*100 URA3*-*1 ADE2*-*1 HIS3*-*11,1)*	
V02	W303, *LEU2::P*_*GAL1*_-*CnVS*-*T*_*CYC1*_	This study
V03	W303, *LEU2::P*_*GAL1*_-*ERG20*-*T*_*CYC1*_	This study
V04	W303, *LEU2::P*_*GAL1*_-*CnVS*-*T*_*CYC1*_*,P*_*GAL1*_-*ERG20*-*T*_*CYC1*_	This study
V05	W303, *LEU2::P*_*GAL1*_-*ERG20*-*GSG*-*CnVS*-*T*_*CYC1*_	This study
V06	W303, *LEU2::P*_*GAL1*_-*ERG20*-*GGGGS*-*CnVS*-*T*_*CYC1*_	This study
V07	W303, *LEU2::P*_*GAL1*_-*ERG20*-*GSGGGGS*-*CnVS*-*T*_*CYC1*_	This study
V08	W303, *LEU2::P*_*GAL1*_–*CnVS*-*GSG*-*ERG20*-*T*_*CYC1*_	This study
V09	W303, *LEU2::P*_*GAL1*_-*CnVS*-*T*_*CYC1*_*, URA3::P*_*TPI1*_-*tHMG1*-*T*_*CYC1*_	This study
V10	W303, *LEU2::P*_*GAL1*_-*CnVS*-*T*_*CYC1*_*_P*_*GAL1*_-*ERG20*-*T*_*CYC1*_*, URA3::P*_*TPI1*_-*tHMG1*-*T*_*CYC1*_	This study
V11	W303, *LEU2::P*_*GAL1*_-*ERG20*-*GSG*-*CnVS*-*T*_*CYC1*_, *URA3::P*_*TPI1*_-*tHMG1*-*T*_*CYC1*_	This study
V13	W303, *LEU2::P*_*GAL1*_-*ERG20*-*GSGGGGS*-*CnVS*-*T*_*CYC1*_*, URA3::P*_*TPI1*_-*tHMG1*-*T*_*CYC1*_	This study
V15	W303,*LEU2::P*_*GAL1*_-*ERG20*-*GSG*-*CnVS*-*T*_*CYC1*_,*URA3::P*_*TPI1*_-*tHMG1*-*T*_*CYC1*_*, PERG9Δ::KanMX*-*P*_*HXT1*_	This study
V16	W303, *LEU2::P*_*GAL1*_-*ERG20*-*GGGGS*-*CnVS*-*T*_*CYC1*_, *URA3::P*_*TPI1*_-*tHMG1*-*T*_*CYC1*_*, PERG9Δ::KanMX*-*P*_*HXT1*_	This study
N05	W303, *LEU2::P*_*GAL1*_-*ERG20*-*GSG*-*CnVS*-*T*_*CYC1*_, *URA3::P*_*TPI1*_-*tHMG1*-*T*_*CYC1*_*,PERG9Δ::KanMX*-*P*_*HXT1*_*, TRP1::P*_*GAL1*_-*ValOx*-*T*_*CYC1*_	This study
N06	W303, *LEU2::P*_*GAL1*_-*ERG20*-*GSG*-*CnVS*-*T*_*CYC1*_, *URA3::P*_*TPI1*_-*tHMG1*-*T*_*CYC1*_*, PERG9Δ::KanMX*-*P*_*HXT1*_*, TRP1::P*_*GAL1*_-*HPO*-*T*_*CYC1*_*_P*_*GAL1*_-*ATR1*-*T*_*CYC1*_	This study
N11	W303, *LEU2::P*_*GAL1*_-*ERG20*-*GSG*-*CnVS*-*T*_*CYC1*_, *URA3::P*_*TPI1*_-*tHMG1*-*T*_*CYC1*_*, PERG9Δ::KanMX*-*P*_*HXT1*_*, TRP1::P*_*GAL1*_-*HPO*-*T*_*CYC1*_*_P*_*GAL1*_-*ATR1*-*T*_*CYC1*_*HIS3::P*_*GAL1*_-*ADH6*-*T*_*CYC1*_	This study
N12	W303, *LEU2::P*_*GAL1*_-*ERG20*-*GSG*-*CnVS*-*T*_*CYC1*_, *URA3::P*_*TPI1*_-*tHMG1*-*T*_*CYC1*_*, PERG9Δ::KanMX*-*P*_*HXT1*_*, TRP1::P*_*GAL1*_-*HPO*-*T*_*CYC1*_*_P*_*GAL1*_-*ATR1*-*T*_*CYC1*_*HIS3::P*_*GAL1*_-*ADH2*-*T*_*CYC1*_	This study
N14	W303, *LEU2::P*_*GAL1*_-*ERG20*-*GSG*-*CnVS*-*T*_*CYC1*_, *URA3::P*_*TPI1*_-*tHMG1*-*T*_*CYC1*_*, PERG9Δ::KanMX*-*P*_*HXT1*_*, TRP1::P*_*GAL1*_-*HPO*-*T*_*CYC1*_*_P*_*GAL1*_-*ATR1*-*T*_*CYC1*_*HIS3::P*_*GAL1*_-*ADH*-*C3*-*T*_*CYC1*_	This study
N15	W303, *LEU2::P*_*GAL1*_-*ERG20*-*GSG*-*CnVS*-*T*_*CYC1*_, *URA3::P*_*TPI1*_-*tHMG1*-*T*_*CYC1*_*, PERG9Δ::KanMX*-*P*_*HXT1*_*, TRP1::P*_*GAL1*_-*HPO*-*T*_*CYC1*_*_P*_*GAL1*_-*ATR1*-*T*_*CYC1*_*HIS3::P*_*GAL1*_-*ZSD1*-*T*_*CYC1*_	This study
N16	W303, *LEU2::P*_*GAL1*_-*ERG20*-*GSG*-*CnVS*-*T*_*CYC1*_, *URA3::P*_*TPI1*_-*tHMG1*-*T*_*CYC1*_*, PERG9Δ::KanMX*-*P*_*HXT1*_*, TRP1::P*_*GAL1*_-*HPO*-*T*_*CYC1*_*_P*_*GAL1*_-*ATR1*-*T*_*CYC1*_*HIS3::P*_*GAL1*_-*ABA2*-*T*_*CYC1*_	This study

Yeast transformations were performed by the standard lithium acetate method. Various metabolic engineered strains were thus constructed by the transformation of the appropriate plasmid into the *S. cerevisiae* W303-1A strain. The replacement of ERG9 promoter was accomplished by transforming the PCR amplified integration cassette into the appropriate strains. Transformants were selected on YNB auxotroph plates or selected for geneticin resistance. The correct integration was confirmed by PCR.

### Flask fermentation for (+)-valencene and (+)-nootkatone production

A pre-culture was prepared by inoculating a single colony into 5 ml auxotroph YNB medium and cultivating O/N at 30 °C, 200 rpm. YPD medium with 0.2% glucose and 2% galactose as carbon sources was used as the following fermentation medium for (+)-valencene and (+)-nootkatone production. The flask fermentations were initiated by inoculating the pre-culture into 50 ml fermentation medium at an OD_600nm_ of 0.01 and cultured at 30 °C, 200 rpm for 90 h. An organic layer of n-dodecane (10%, v/v) was added to extract products from the fermentation medium. The cultures were centrifuged at 8000 rpm for 10 min. The n-dodecane phase was sampled and stored at 4 °C for further analysis.

### Product analysis by GC-FID and GC–MS

Samples (500 μl) were mixed with 1 ml of n-hexane and 1 μl of the mixed sample was injected to be analyzed by GC-FID or GC–MS on a QP2010 instrument (Shimadzu). The production of (+)-valencene was analyzed using an rtx-1 column. The initial temperature was maintained at 40 °C for 2 min and the temperature program of 40–160 °C at 10 °C/min and 160–250 °C at 15 °C/min were followed. The production of β-nootkatol and (+)-nootkatone was analyzed using an rtx-1701 column and a temperature program of 70–200 °C at 10 °C/min and 200–280 °C at 30 °C/min. The produced farnesol was analyzed using an rtx-1701 column and a temperature program of 40–180 °C at 25 °C/min and 180–250 °C at 15 °C/min. The MS spectra of valencene, β-nootkatol, (+)-nootkatone, and farnesol were compared with those of authentic standards. The amounts of valencene, (+)-nootkatone, and farnesol were determined by GC-FID with the calibration curve generated by their respective standards. Due to the lack of standard, the amount of β-nootkatol in different strains was represented with their peak surface areas or their relative percentages.

### Effects of DMSO and Triton X-100 on the production of (+)-nootkatone

Due to the high volatility and low solubility of (+)-valencene, it is easily extracted by the added n-dodecane and thus the intracellular (+)-valencene concentration becomes very low, which is unfavorable for further (+)-nootkatone synthesis. In order to increase the solubility of (+)-valencene in aqueous phase, different concentrations of DMSO (1%, 2%, 4%, and 8%, v/v) and Triton X-100 (0.02%, 0.06%, and 0.1%, v/v) were added to the fermentation medium with 1/10 of n-dodecane. The growth of N06 strain was determined by measuring OD_600nm_. The production of (+)-nootkatone in the n-dodecane phase were measured by GC-FID analysis.

### Growth assay and the effect of n-dodecane or (+)-valencene on growth

The growth of metabolic engineered *S. cerevisiae* strains was determined by measuring the optical density at OD_600nm_. The pre-cultures of *S. cerevisiae* strains were inoculated in 50 ml YPD with different concentrations of n-dodecane and (+)-valencene at OD_600nm_ of 0.1 and their growth was monitored by measuring the optical density at OD_600nm_ for 28 h. The cells were harvested by centrifugation and washed two times with water. The dry cell weight (DCW) was obtained by measuring the weight after freeze drying.

## Supplementary information

**Additional file 1.** Additional figures.

## Data Availability

All data generated or analysed during this study are included in this published article and its additional files.

## Abbreviations

CPR: Cytochrome P450 reductase
DMAPP: Dimethylallyl pyrophosphate
ERG9: Squalene synthase enzyme
ERG20: Diphosphate synthase
FPP: Farnesyl diphosphate
GPP: Geranyl pyrophosphate
HPO: *Hyoscyamus muticus* premnaspirodiene oxygenase
IPP: Isopentenyl diphosphate
MEP: Methyl-d-erythritol phosphate pathway
MVA: Mevalonate pathway
SDR: Short-chain dehydrogenase/reductase
tHMG1: Truncated form of 3-hydroxy-3-methylglutaryl-CoA (HMG-CoA) reductase
