# Supplementary material for: Metabolic engineering Saccharomyces cerevisiae for de novo production of the sesquiterpenoid (+)-nootkatone
Source: Microb Cell Fact. 2020 Feb 3;19:21. doi: 10.1186/s12934-020-1295-6 (PMC6998195; doi:10.1186/s12934-020-1295-6)
Supplement: Supplementary file 1 — Additional file 1. Additional figures. [file 12934_2020_1295_MOESM1_ESM.pdf]

Additional file

Metabolic engineering *Saccharomyces cerevisiae* for de novo production of the sesquiterpenoid (+)-nootkatone

Xiangfeng Meng, Hui Liu, Wenqiang Xu, Weixin Zhang, Zheng Wang, Weifeng Liu*

State Key Laboratory of Microbial Technology, Shandong University, No.72 Binhai Road, Qingdao 266237, P. R. China

*Correspondence should be addressed to W Liu. E-mail: weifliu@sdu.edu.cn

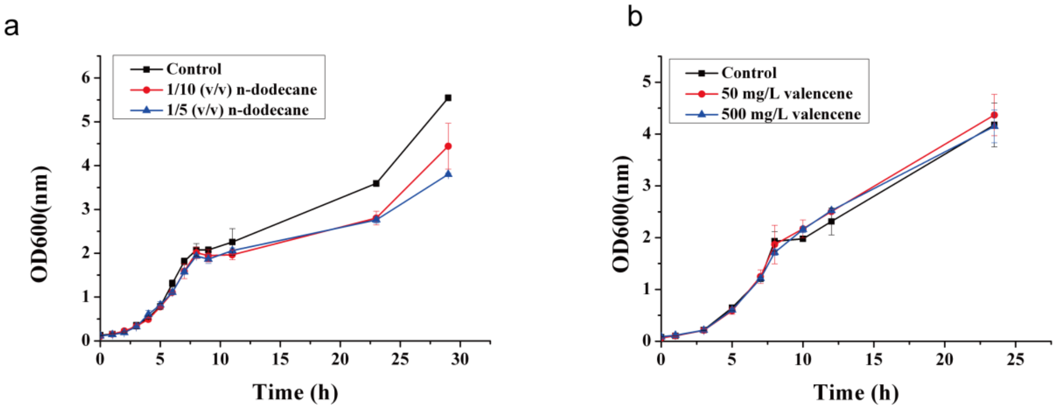

Figure S1. The effect of n-dodecane and (+)-valencene on the growth of *S. cerevisiae*. Different concentrations of n-dodecane (v/v) (a) and (+)-valencene (mg/L) (b) were added to the culture media and the growth of *S. cerevisiae* was monitored by measuring the optical density at OD_{600nm}.

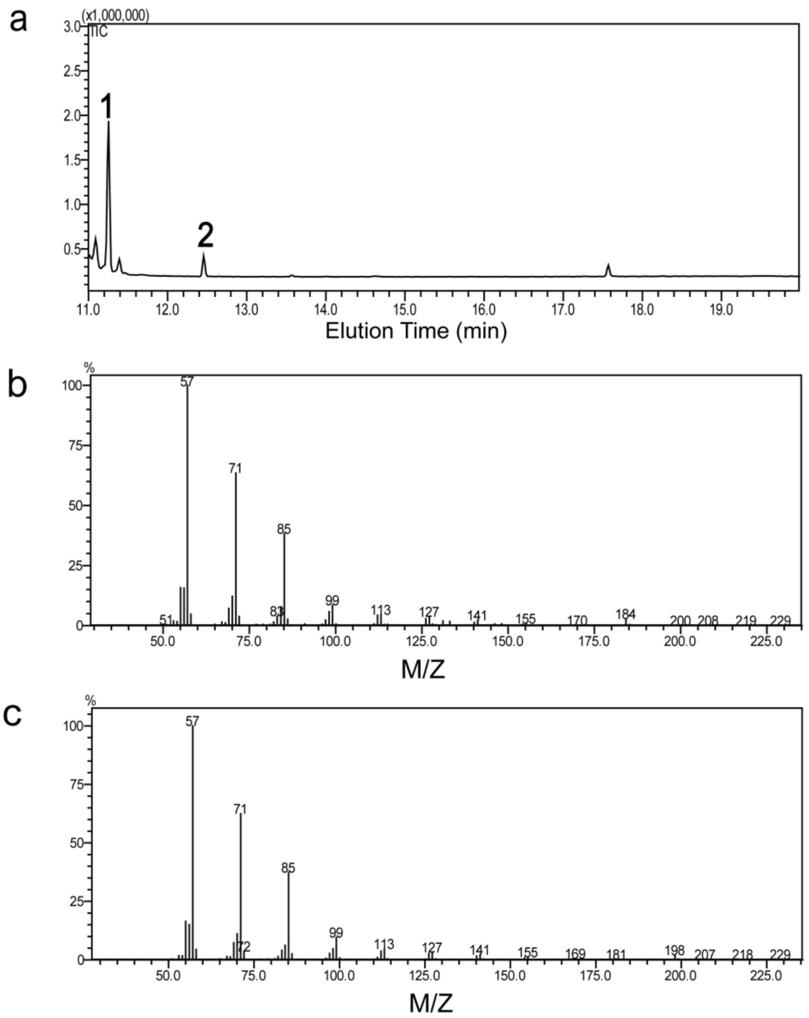

Figure S2 GC-MS analysis of solvent control. (a): The GC-MS profiles of solvent control (1:2 v/v n-dodencane: n-hexane); (b): Mass spectrum of n-tridecane (peak 1).; (c): Mass spectrum of n-tetradecane (peak 2).

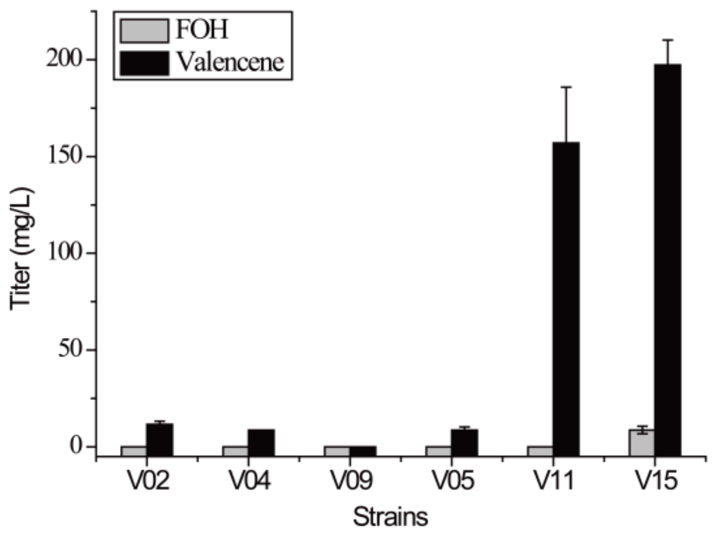

Figure S3. Farnesol and (+)-valencene production in different metabolically engineered *S. cerevisiae* strains.

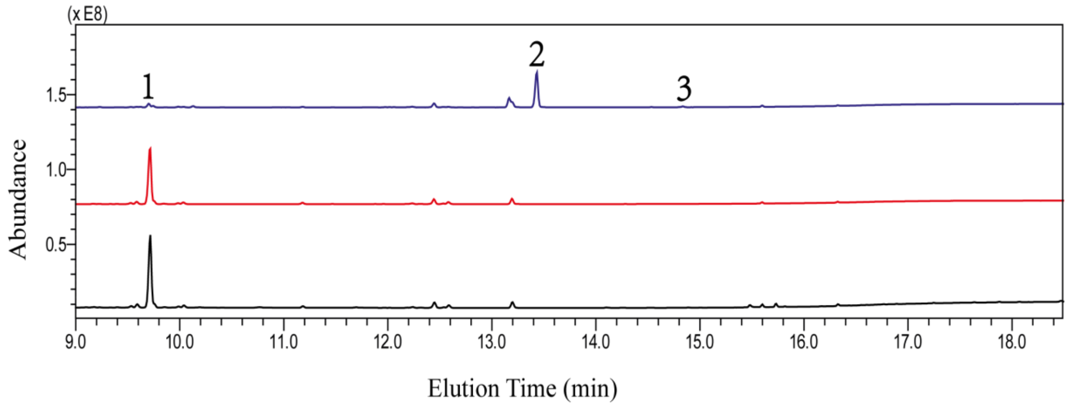

Figure S4. GC-MS analysis of the extracted n-dodecane phase of strain V15 (black), strain N05 (red) and strain N06 (blue) shake-flask culture fermentations. Strain V15 produced significant amount of (+)-valencene (peak 1). The overexpression of HPO and ATR1 (strain N06) gave rise to β-nootkatol (peak 2) as the predominant product and (+)-nootkatone (peak 3) as a minor product while the (+)-valencene reduced significantly. However, ValOx overexpression (strain N05) did not produce β-nootkatol and (+)-nootkatone.

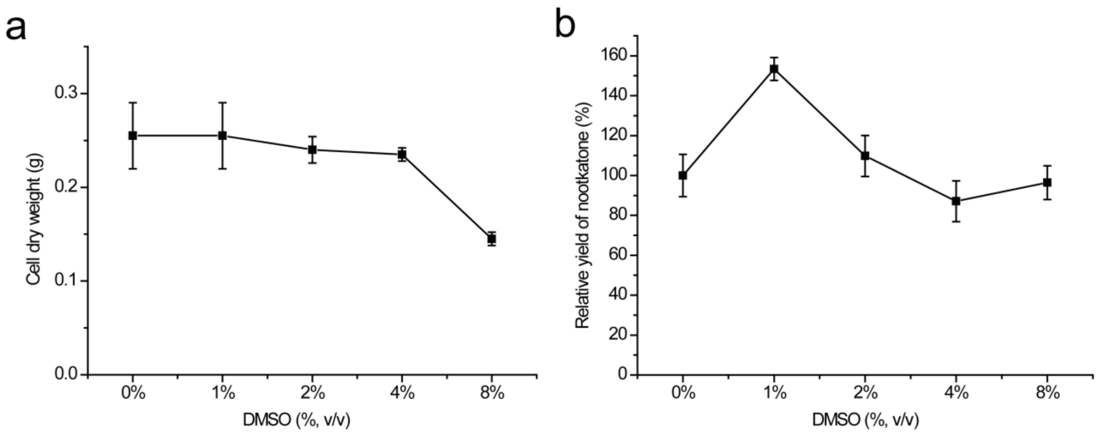

Figure S5. The effect of adding DMSO to the fermentation media on the growth (a), and (+)-nootkatone yield (b) of the *S. cerevisiae* N06 strain. The growth of N06 was determined by measuring the cell dry weight after freeze drying while the yield of (+)-nootkatone were shown as the relative yield to those of N06 cultured without DMSO.

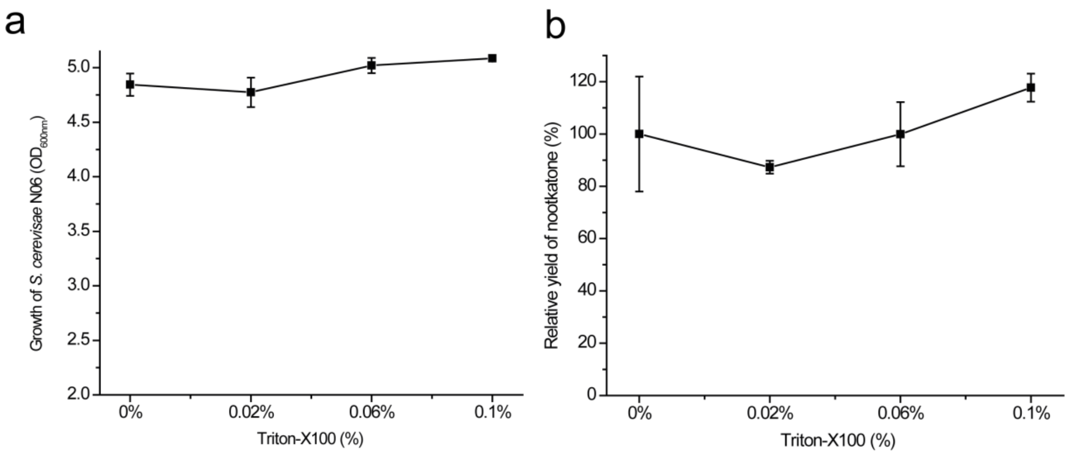

Figure S6. The effect of adding Triton-X100 to the fermentation media on the growth (a) and (+)-nootkatone yield (b) of the *S. cerevisiae* N06 strain. The growth of N06 was determined by measuring the optical density at OD_{600nm} while the yield of (+)-nootkatone were shown as the relative yield to those of N06 cultured without Triton-X100.

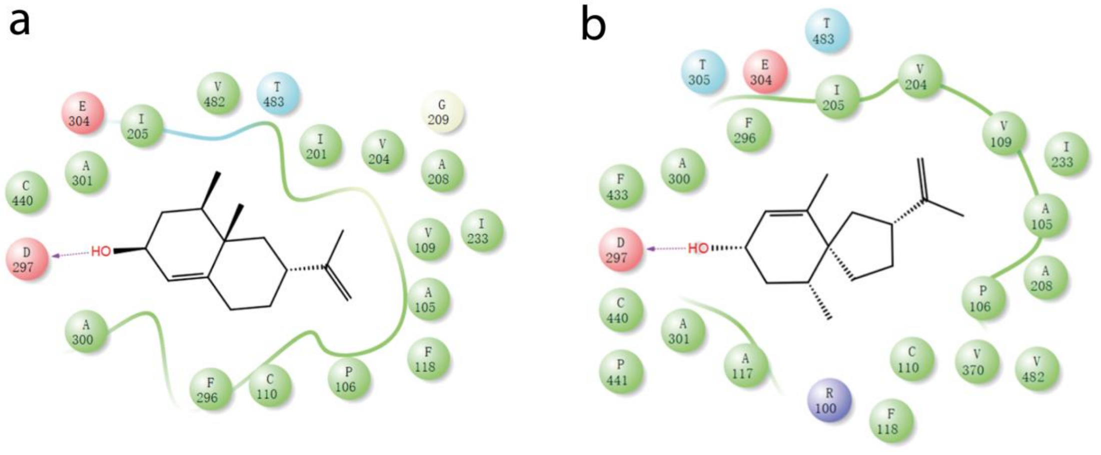

Figure S7. Predicted binding sites for ligands bound to HPO: (a) β-nootkatol and (b) solavetivol. Residues within 5 Å of each ligand are shown on the corresponding 2D ligand interaction diagram. The 3D modeled structure of HPO was firstly constructed by threading the HPO amino acid sequence (GenBank accession number EF569601.1) onto the structural coordinates for the Cytochrome P450 (PDB code 6B82). Then we docked ligands β-nootkatol and solavetivol to the active binding site of HPO. The predicted HPO/β-nootkatol and HPO/solavetivol complexes were then embedded in water environment and relaxed using molecule dynamic simulation for 5ns. MM/GBSA computational approach was used to calculate the binding free energy of the ligand and decompose the free energy at the amino acid level. Both of these two ligands form H-bond with D297 and hydrophobic interaction mainly with F118, I205, F296, A300, and V482. Notably, the calculated binding free energy of solavetivol (-25.1±2.5 kcal/mol) is lower than that of β-nootkatol (-22.7±3.7 kcal/mol). This difference may not be enough to account for the high efficiency catalysis of solavetivol, which is somehow able to establish more favorable interactions with HPO than β-nootkatol.

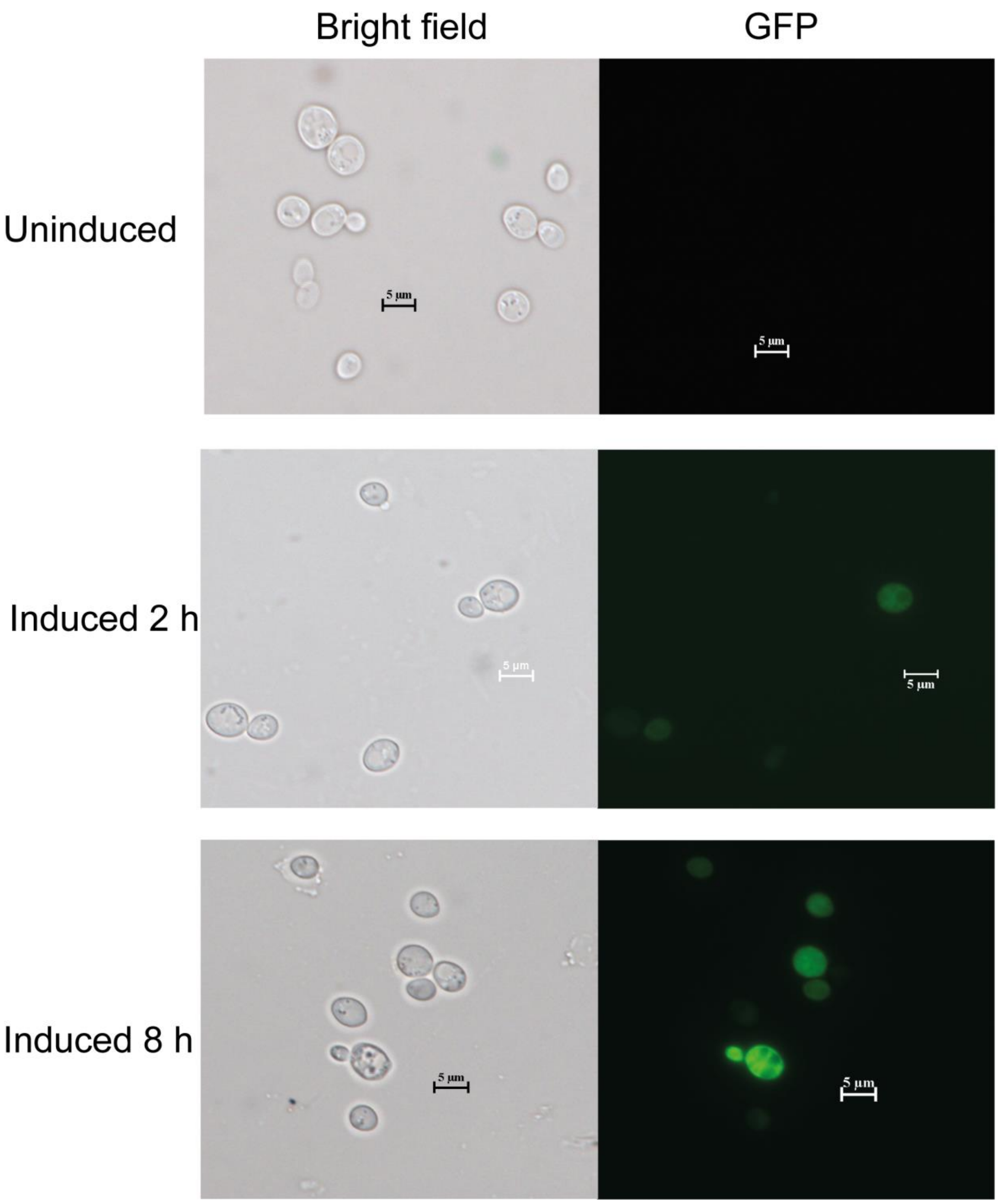

Figure S8. Fluorescence detection of the expression of ValOx-GFP induced with 2% galactose. ValOx was expressed in the recombinant *S. cerevisiae* strain by fusing GFP to the C-terminus of ValOx.
